# Fullerenemalonates inhibit amyloid beta aggregation, *in vitro* and *in silico* evaluation

**DOI:** 10.1039/c8ra07643j

**Published:** 2018-11-27

**Authors:** Martínez-Herrera Melchor, Figueroa-Gerstenmaier Susana, García-Sierra Francisco, Beltrán Hiram I., Rivera-Fernández Norma, Lerma-Romero Jorge A., López-Camacho Perla Y., Basurto-Islas Gustavo

**Affiliations:** CONACYT, Metropolitan Autonomous University Cuajimalpa Mexico City 05300 Mexico; Department of Natural Sciences, Metropolitan Autonomous University Cuajimalpa Mexico City 05300 Mexico; Department of Chemical, Electronic & Biomedical Engineering, Division of Sciences and Engineering, University of Guanajuato Loma del Bosque No.103, Lomas del Campestre León 37150 Guanajuato Mexico gustavo.basurto@ugto.mx; Eduard-Zintl-Institut für Anorganische und Physikalische Chemie, Technische Universität Darmstadt D-64287 Darmstadt Germany; Department of Cell Biology, Center of Research and Advanced Studies of the National Polytechnic Institute (CINVESTAV) Mexico City 07360 Mexico; Department of Microbiology and Parasitology, School of Medicine, National Autonomous University of Mexico Ciudad de México 04510 Mexico; National Polytechnic Institute Mexico City 07738 Mexico

## Abstract

The onset of Alzheimer's disease (AD) is associated with the presence of neurofibrillary pathology such as amyloid β (Aβ) plaques. Different therapeutic strategies have focused on the inhibition of Aβ aggregate formation; these pathological structures lead to neuronal disorder and cognitive impairment. Fullerene C_60_ has demonstrated the ability to interact and prevent Aβ fibril development; however, its low solubility and toxicity to cells remain significant problems. In this study, we synthesized, characterized and compared diethyl fullerenemalonates and the corresponding sodium salts, adducts of C_60_ bearing 1 to 3 diethyl malonyl and disodium malonyl substituents to evaluate the potential inhibitory effect on the aggregation of Aβ_42_ and their biocompatibility. The dose-dependent inhibitory effect of fullerenes on Aβ_42_ aggregation was studied using a thioflavin T fluorescent assay, and the IC_50_ value demonstrated a low range of fullerene concentration for inhibition, as confirmed by electron microscopy. The exposure of neuroblastoma to fullerenemalonates showed low toxicity, primarily in the presence of the sodium salt-adducts. An isomeric mixture of bisadducts, trisadducts and a *C*_3_-symetrical trisadduct demonstrated the highest efficacy among the tests. *In silico* calculations were performed to complement the experimental data, obtaining a deeper understanding of the Aβ inhibitory mechanism; indicating that *C*_3_-symetrical trisadduct interacts mainly with 1D to 16K residues of Aβ_42_ peptide. These data suggest that fullerenemalonates require specific substituents designed as sodium salt molecules to inhibit Aβ fibrillization and perform with low toxicity. These are promising molecules for developing future therapies involving Aβ aggregates in diseases such as AD and other types of dementia.

## Abstract 2

Synthesis of new non toxic nanomaterials, with high anti-amyloid fibrils formation effect, *in vitro* and *in silico*.

## Graphical abstract

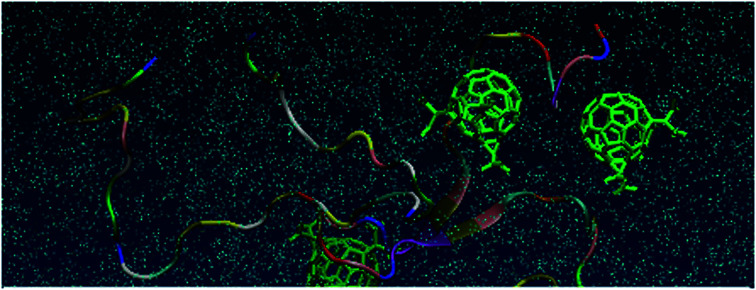

## Introduction

Amyloid plaques are the primary hallmark of Alzheimer's disease (AD), the most prevalent type of dementia worldwide. The aggregation of the amyloid beta (Aβ) peptide leads to the onset of extracellular plaque throughout the cortical mantle. The β- and γ-secretases sequentially proteolyze the amyloid precursor protein (APP), releasing a 40 or 42 amino acid peptide. In AD, Aβ aggregates extracellularly form soluble oligomers, insoluble β-sheet protofibrils, fibrils and plaques. Aβ plays an important role in the onset of AD, described in the amyloid cascade hypothesis, resulting from a chronic imbalance between Aβ production and Aβ clearance^1^ that turns into: neuronal loss, neurofibrillary tangle formation, vascular damage, and dementia that correlates directly to Aβ deposition. Despite Aβ plaques showing a low correlation with dementia, Aβ oligomers display high toxicity to neurons^2,3^ suggesting that Aβ fibrillogenesis plays an important role in AD-induced toxicity. The Aβ aggregation involves the C-terminus of the peptide that determines the rate of fibril formation while the N-terminus promotes Aβ–Aβ interaction for polymerization, leading to a random coil or α-helix to β-sheet transition *via* a nucleation mediated process.^4^ The extended β-sheets promote homophilic interactions and eventually lead to Aβ oligomer formation. Kinetic studies demonstrated the monomeric Aβ requirement for oligomer formation as seeds/nuclei, rich in β-sheets, for accelerated fibril growth.^5^

Several strategies for targeting Aβ production and clearance have failed, since Aβ immunotherapies induced encephalomyelitis and possible microhemorrhages,^6,7^ and the inhibition of secretases disrupts important metabolic processes.^8^ Therefore, another strategy is based on inhibition of the Aβ peptide self-assembly. Dyes and small molecules,^9–11^ peptides^12–14^ and nanoparticles^15–18^ were identified as effective inhibitors of Aβ aggregation, ameliorating cell survival and cognitive deficit.

Fullerene C_60_, a carbon nanomaterial with a symmetric nanostructure, has been extensively used in areas of science, particularly in biomedics. Even though it has great potential in biological applications, the solubility of fullerenes has shown low compatibility in biological systems because of its hydrophobicity; so different strategies have been developed to achieve soluble fullerenes in aqueous dispersions for biological applications, including as an inhibitor of human immunodeficiency virus-1 (HIV-1) protease,^19^ an antioxidant,^20^ and a cancer therapeutic.^21^ C_60_ functions as both a reactive oxygen species (ROS) producer under UV or visible light, and a ROS scavenger in the dark. This dual property of fullerenes to either quench or generate cell-damaging ROS has been applied as a cytoprotective or cytotoxic anticancer/antimicrobial/anti-Aβ agent.^22–26^ Previous reports indicated that fullerenes, and certain fullerene derivatives, inhibit Aβ aggregation much more efficiently under photo-irradiation with visible light.^24,25^ The dual property of C_60_ to either scavenge or produce ROS has been used for a synergistic therapy for Alzheimer's disease (AD).^26^

Fullerenes have two important advantages in AD research: their structure allows them to cross the blood–brain barrier^27^ and they show a high capacity to inhibit Aβ fibril formation.^16,17,24,25,28–30^ However, there is controversy regarding the biocompatibility of fullerenes: some groups report nontoxic effects in different tested models, such as the LLC-PK1 proximal tubule cell model^31^ or L929 mouse subcutaneous connective cells;^32^ however, a high concentration of fullerenes induces toxicity in the same studies. Therefore, the correlation between the amount of fullerene necessary to perform its biological activity and biocompatibility remains unclear. Recently, impaired spatial memory with a significant decrease in BDNF protein levels and gene expression has been demonstrated in rats injected with C_60_, but in contrast, it showed high antioxidant capacity.^33^ Fullerene might be an important molecule for the treatment of neurodegenerative disorders, but the molecular design requires further research. An interesting approach to improve fullerenes for AD treatment is based on functionalization with different substituents that promote its stability, biocompatibility, capacity to cross the blood–brain barrier and ability to inhibit Aβ fibril formation. One successful method to obtain more polar fullerenes introducing functional groups consists in exohedral functionalization by the Bingel reaction.^34,35^ In particular, functionalization with a malonate group led to water-soluble fullerenes and in addition serves as a precursor for further functionalization, so it can be used to link a variety of functional groups to obtain different derivatives.

**Fig. 1:**
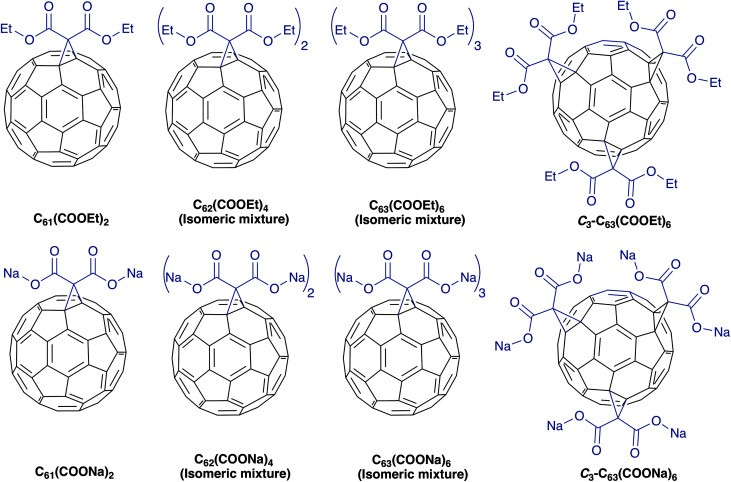
Molecular structure of diethyl fullerenemalonates and the corresponding sodium salts, synthesized and evaluated in this study.

In this study we synthesized diethyl fullerenemalonates and the corresponding sodium salts, through the Bingel reaction to obtain adducts of C_60_ holding 1 to 3 diethyl malonyl and disodium malonyl substituents (C_60+*n*_(COOR)_2*n*_, where *n* = 1–3 and R = –CH_2_CH_3_, –Na) (Fig. 1). This is the first report to demonstrate the effect of the type and number of organic addends of fullerenemalonates on anti-Aβ activity and their biocompatibility with cells. The potential inhibitory effect of the fullerenes on Aβ_42_ fibril formation was shown by thioflavin fluorescence assay and electron microscopy. Low cytotoxicity was shown in neuroblastoma SH-SY5Y cells exposed to the fullerenemalonates during 24 h, and the cytotoxic effect decreased even more in the presence of the corresponding sodium salt molecules. The *in silico* data obtained by atomistic molecular dynamics showed that the purified *C*_3_ trisadduct binds to the Aβ_42_ monomer, mostly to 1D, 5R, 16K residues by means of hydrogen bonds and to 2A, 4F, 6H, 8S, 12V, 15Q residues.

## Materials and methods

### Chemicals and reagents

Fullerene C_60_ 98%, diethyl malonate 99%, carbon tetrabromide 99%, 1,8-diazabicyclo[5.4.0]-undec-7-ene (DBU), 98%, thioflavin T (ThT), and all other chemicals and solvents were purchased from Sigma-Aldrich and were used as supplied without further purification. Beta-amyloid (1–42) Human was purchase from (ANASPEC-1 mg).

### Fullerene synthesis

The Bingel-type adducts were synthesized by cyclopropanation of C_60_ with different equivalents of diethyl malonate, CBr_4_ and 1,8-diazabicyclo-[5,4,0]undec-7-ene (DBU) as an auxiliary base, following the procedure published by X. Camps *et al.* and E. Straface *et al.*^36,37^ Diethylmalonate mono- and bisadducts were isolated and purified by a chromatography column on silica gel. Chromatographic separation of the reaction mixture with *n*-hexane/toluene (65 : 35) generated a first fraction consisting of a residual amount of C_60_. Subsequently, pure toluene was used to yield a second fraction that consisted of the monoadduct contaminated with traces of C_60_, and finally there was a third fraction containing an isomeric mixture of the corresponding bisadducts. Seven regioisomers of the bisadducts (of the eight possible) have been isolated and characterized by Hirsch *et al.*^38^ The second and third fractions were also purified by chromatography, using *n*-hexane/toluene (65 : 35) and pure toluene to remove the traces of C_60_ or monoadduct to obtain pure monoadduct, C_61_(COOEt)_2_, and an isomeric mixture of bisadducts, C_62_(COOEt)_4_. The trisadducts were purified using elution of the reaction mixture with *n*-hexane/toluene (65 : 35) to pure toluene, allowing the separation of three fractions containing enriched samples of monoadduct, bisadducts and trisadducts as the major components, respectively. The third fraction containing semipure trisadducts was rechromatographed on silica gel using toluene as the mobile phase to obtain the purified isomeric mixture, C_63_(COOEt)_6_. Seven regioisomers of the trisadducts (of the 10 possible considering the restriction that only *e* or *trans* additions to bisadducts having *e*- and *trans-positional* relationships are considered) have been isolated and characterized by Djojo *et al.*^39^ A purified sample of the C_3_ trisadduct (*C*_3_-C_63_(COOEt)_6_) was obtained by elution of the fraction containing the isomeric mixture of trisadducts with toluene/acetonitrile (99.5 : 0.5), producing a last fraction (red band) enriched in the regioisomeric trisadduct with *C*_3_ symmetry. The fraction containing semipure isomer *C*_3_ was further rechromatographed twice using toluene/acetonitrile (99.5 : 0.5) thus obtaining a pure *C*_3_ adduct (*C*_3_-C_63_(COOEt)_6_). The identity of all compounds was confirmed by electrospray ionization time-of-flight mass spectrometry (ESI-TOF-MS), giving molecular ions identical to those calculated. The obtained data was as follow: C_61_(COOEt)_2_ (*m*/*z*) 878.059 (M^−^), calculated for C_67_H_10_O_4_ 878.058; C_62_(COOEt)_4_ (*m*/*z*) 1036.117 (M^−^), calculated for C_74_H_20_O_8_ 1036.116; C_63_(COOEt)_6_ (*m*/*z*) 1195.181 (M^+^ + 1), calculated for C_81_H_31_O_12_ 1195.181; *C*_3_-C_63_(COOEt)_6_ (*m*/*z*) 1195.179 (M^+^ + 1), calculated for C_81_H_31_O_12_ 1195.181. The samples were further characterized by ^1^H nuclear magnetic resonance (NMR), except for C_62_(COOEt)_4_ and C_63_(COOEt)_6_, ultraviolet visible (UV-vis) and infrared (IR) spectroscopies.

### Diethyl fullerenemalonates

C_61_(COOEt)_2_: ^1^H NMR (400 MHz, CS_2_-CDCl_3_), *δ* 4.54 (q, *J* = 7 Hz, 4H), 1.52 (t, *J* = 7 Hz, 6H); UV-vis (THF) *λ*_max_/nm 327, 427, 477; IR (KBr)/cm^−1^ 2963, 2924, 2853, 1743, 1633, 1538, 1461, 1427, 1385, 1364, 1292, 1262, 1233, 1206, 1179, 1095, 1059, 1019, 860, 804, 733, 705, 670, 579, 540, 525. C_62_(COOEt)_4_: UV-vis (THF) *λ*_max_/nm 303, 417, 428, 474; IR (KBr)/cm^−1^ 2962, 2926, 2854, 1743, 1634, 1538, 1459, 1440, 1386, 1366, 1294, 1232, 1180, 1098, 1059, 1018, 857, 806, 733, 704, 668, 623, 574, 524. C_63_(COOEt)_6_: UV-vis (THF) *λ*_max_/nm 303, 402 (sh), 412 (sh), 462 (w); IR (KBr)/cm^−1^ 2953, 2929, 2900, 2860, 1744, 1632, 1461, 1443, 1388, 1367, 1296, 1232, 1180, 1101, 1063, 1019, 859, 808, 755, 734, 705, 668, 573, 547, 525. *C*_3_-C_63_(COOEt)_6_: UV-vis (THF) *λ*_max_/nm 303, 378 (sh), 390 (sh), 474 (w), 562 (sh); IR (KBr)/cm^−1^ 2962, 2923, 2854, 1743, 1653, 1634, 1462, 1446, 1387, 1369, 1281, 1241, 1214, 1174, 1099, 1064, 1022, 860, 807, 738, 707, 667, 626, 563, 525. All the samples of C_60_ adducts showed identical spectroscopic data to those already reported.^34,40–42^

The disodium fullerenemalonates were synthesized by hydrolysis of the respective diethyl fullerenemalonates, with a 1.5-fold (relative to ester groups) molar amount of NaOH (1 M) in tetrahydrofuran : methanol : water for the case of monoadduct C_61_(COONa)_2_ and bisadducts C_62_(COONa)_4_ and toluene : methanol : water in the case of the trisadducts C_63_(COONa)_6_ and *C*_3_-C_63_(COONa)_6_, according to a method described already.^43^ The reaction was stopped after all of the starting diethyl malonate was consumed, as monitored by thin-layer chromatography. The sodium salts were triturated from toluene and water or methanol and isolated by centrifugation. Finally, the product was evaporated, and dried under vacuum. The yields were nearly quantitative. The samples were characterized by UV-vis and IR spectroscopies. Disodium fullerenemalonates were identified by the shift in the characteristic and strong 〉C=O vibration in the IR spectrum (KBr pellet) of the ester from *v* = 1743 cm^−1^ to *v* = 1638 cm^−1^, 1621 cm^−1^, 1626 cm^−1^ and 1638 cm^−1^ for the monoadduct, isomeric mixture of bisadducts and trisadducts, and *C*_3_-trisadduct, respectively; due to the mesomeric weakening of the 〉C=O double bond in the dicarboxylate.

### Disodium fullerenemalonates

C_61_(COONa)_2_: UV-vis (THF) *λ*_max_/nm 318; IR (KBr)/cm^−1^ 2955, 2926, 2852, 1726, 1638, 1560, 1457, 1407, 1383, 1097, 1066, 1027, 841, 704, 674, 615, 529. C_62_(COONa)_4_: UV-vis (CH_3_OH) *λ*_max_/nm 349; IR (KBr)/cm^−1^ 2960, 2926, 2855, 1728, 1621, 1408, 1376, 1330, 1245, 1204, 1166, 1101, 1066, 841, 704, 649, 613, 526. C_63_(COONa)_6_: UV-vis (CH_3_OH) *λ*_max_/nm 414; IR (KBr)/cm^−1^ 2961, 2928, 2855, 1728, 1626, 1458, 1379, 1331, 1105, 1058, 840, 703, 671, 623, 522. *C*_3_-C_63_(COOEt)_6_: UV-vis (CH_3_OH) *λ*_max_/nm 414; IR (KBr)/cm^−1^ 2959, 2928, 2858, 1728, 1638, 1600, 1580, 1562, 1461, 1410, 1382, 1289, 1272, 1122, 1071, 1039, 961, 848, 796, 742, 702, 651, 602.

### Preparation and fibrillization of Aβ_42_ peptide

The Aβ_42_ monomer (1 mg) was resuspended in 1 ml of cold 1,1,1,3,3,3-hexafluoro-2-propanol (HFIP), and it was kept in the dark for 30 min at 4 °C to solubilize the peptide in the monomeric stage.^44,45^ The solution was aliquoted (25 μl) and dried under vacuum in a rotary evaporator for 1 h at room temperature; the resultant transparent film was stored at −80 °C for further experiments. The amyloid fibrils were formed as previously described;^46^ briefly, the solubilized Aβ_42_ was dissolved in polymerization buffer containing PBS 1× (final concentration of 10 mM PO_4_^3−^, 137 mM NaCl, and 2.7 mM KCl) and 1% DMSO and incubated at 37 °C at different time points. The required volume of polymerization buffer was adjusted according to each experiment.

### Fibril formation analysis by western blot

Western blots were performed using 16% SDS-PAGE, loading 1.5 μg of non-boiled Aβ_42_ fibril samples per line, followed by transfer to a nitrocellulose membrane and blocking with 5% skim milk. The blot was performed with a mouse monoclonal anti-β-amyloid antibody (Sigma-Aldrich) at 1 : 3000 dilution, incubated overnight and probed with goat anti-mouse Ig-G (Millipore) at 1 : 10 000, and, finally, detected using enhanced chemiluminescence reagents (Thermo Scientific).

### Fluorescence analysis

Fibrillization of Aβ_42_ peptide was detected by ThT fluorescence intensity that correlates with the number of fibrils formed.^47^ ThT was added to the Aβ_42_ samples to final concentrations of 10 and 20 μM, respectively, in a total volume of 200 μl of polymerization buffer. The fluorescence was measured in a 96-well plate during 24 h at 37 °C using a TECAN Infinite (M1000PRO) spectrofluorometer at 440 nm excitation and 490 nm emission. The fluorescence intensity was the normalized value of Aβ_42_ aggregates at 24 h (positive control). Likewise, to evaluate the inhibitory effect of the fullerenes, 13 μM (final concentration in the solution) of the respective fullerene was added to the same reaction. The relative fluorescence of fullerenemalonates without Aβ_42_ was measured and subtracted from the respective assay with the peptide. The IC_50_ for Aβ_42_ amyloid fibrillization inhibition were determined from the curves obtained by fitting the average fluorescence values in three independent experiments at the following fullerene concentrations: 2.5, 5, 7.5 and 10 μM. The experiments were done in triplicate.

### Electron microscopy

The polymerization assay solution samples and that with disodium fullerenemalonates *C*_3_-C_63_(COONa)_6_ and an isomeric mixture of C_62_(COONa)_4_ were sedimented and placed side by side onto Formvar-coated copper grids for 1 min, followed by incubation in 50 mM ammonium bicarbonate for carbon coating of the sample for 3 min and then negatively stained with 2% uranyl acetate for 1 min. This procedure was repeated twice for 2 min and 1 min, respectively. After drying, the samples were imaged with a JEOL 1400 EX transmission electron microscope (TEM). The experiment was done in duplicate.

### Cell viability assay

The neuroblastoma cell lines SH-SY5Y (ATCC) were grown in 25 cm^2^ flasks at 37 °C with 5% CO_2_ in advanced DMEM/F-12 medium (Sigma-Aldrich), supplemented with 10% fetal bovine serum (Invitrogen); for cytotoxicity assay, cells were seeded at a density of 1 × 10^4^ in triplicate per sample in a 96 well microplate 24 h before the treatment. The fullerenemalonates were incubated for 24 h at the corresponding concentration for their ability to inhibit Aβ_42_ aggregation, 13 μM. Likewise, the vehicles of each fullerenemalonate other than water (acetonitrile and ethanol) were assessed to validate its toxicity. The cell viability assay was evaluated using Thiazolyl Blue Tetrazolium Bromide (MTT, Sigma-Aldrich); based on the conversion of MTT to water-insoluble MTT-formazan of dark blue color by the mitochondrial dehydrogenases of living cells. The absorbance of formazan was measured at a wavelength of 570 nm in a plate reader, iMark Biorad. For a cytotoxic concentration (CC_50_) the fullerenemalonates were incubated at different concentrations followed by a cell viability assay. The CC_50_ was determined from the curves obtained by fitting the average absorbance values in three independent experiments in the 10–80 μM fullerenemalonate concentration range.

### *In silico* experiments

The topology, pdb file, for the Aβ_42_ molecule was taken from Crescenzi O. *et al.*;^48^ the derivative fullerene was obtained by editing a pdb topology file of trisadduct with a diethyl malonyl substituents,^49^ and further optimized using the Automated Topology Builder.^50^ The atomistic force field used was CHARMM36 ^51^ together with the TIP3P model for water^52^ modified^53^ for use with this specific force field. Four systems were considered: one simulation of Aβ_42_ chains *in vacuo*, two of Aβ_42_ in water at two different temperatures and the last one was composed of Aβ_42_ chains with trisadducts of fullerene, in water. The amount of water added was enough to mimic the experimental value of the water density under thermodynamic conditions of pressure and temperature. In the case of the ternary system, the concentration of Aβ_42_ and fullerene was kept low, close to the experimental values, preventing an excess of water molecules. The details of the simulation boxes and thermodynamic conditions are shown in Table 1. The pure system and the binary systems were used to set the stability of the peptide structure when we use the force field CHARMM36. The initial boxes were prepared in an ensemble with a constant number of particles, constant pressure and constant temperature (NPT), using a Berendsen thermostat and barostat; the conditions were then changed to use an NVT ensemble, where *V*, the volume of the simulation box, is kept fixed, using a Nose–Hoover thermostat and turning off the barostat, setting the volume of the box to the average value obtained in the equilibrated NPT simulation. After equilibration was reached (around 100 ns), the simulations were sampled for 10 ns. The Verlet algorithm was used to integrate the movement equations with a time step of 0.001 ps. Long-range electrostatic interactions were calculated using the Particle Mesh Ewald method with 1.2 nm as the cut-off. The van der Waals interactions were calculated using a cut-off equal to 1.2 nm. The coupling times of temperature and pressure were fixed to 2.0 ps.

**Table 1:** Molecular dynamics simulation details and thermodynamic conditions

System	Box type	Box volume (nm^3^)	Temperature (K)	Pressure (bar)	N water	N Aβ	N fullerene
(1) Pure *in vacuo*	Rhombic dodecahedron	422.9	310.15	1	0	64	
(2) In water	Rhombic dodecahedron	11 941.5	310.15	1	389 005	64	
(3) In water	Rhombic dodecahedron	12 214.6	333.15	1	389 005	64	
(4) Ternary	Cubic	679.34	310.15	1	21 250	8	6

## Results

### Aβ polymer formation

**Fig. 2:**
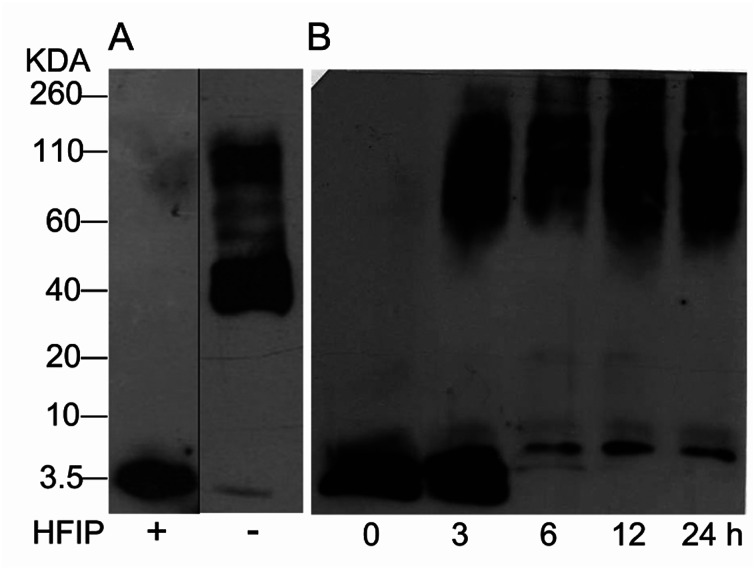
Western blot analysis from Aβ_42_ monomers resuspended or not with HFIP, panel (A). Aβ_42_ polymerization assay for 24 h at 37 °C (B).

The study of Aβ aggregation inhibition requires an assay with Aβ monomers that interact with molecules that prevent further aggregation. To validate that Aβ monomers without pre-aggregated formation were appropriate for evaluating the adducts of C_60_, we used HFIP that breaks the beta sheet structures, preventing Aβ aggregation. The monomer and fibril formation were observed by western blot. The Aβ monomer without pre-aggregates is shown in Fig. 2A with a single band between 2 and 4 kDa, and aggregates between 40 and 160 kDa were formed under the same conditions except for HFIP treatment, demonstrating the requirements of the treatment. Moreover, we evaluated fibril formation during 24 h. Following 3 h of polymerization, low and high molecular weight polymers were seen as well as a reduction in the monomer at 6 h, validating the efficacy of the assay (Fig. 2B).

### Fullerenemalonates inhibit amyloid β peptide aggregation

**Fig. 3:**
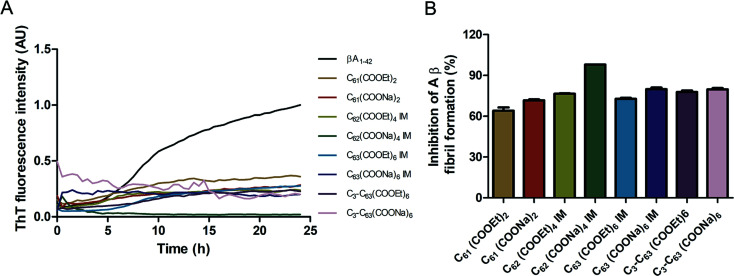
Fullerenemalonates inhibit amyloid β peptide aggregation, analysed by ThT assay during 24 h (A), comparison at 24 h as a percentage of inhibition (B).

**Fig. 4:**
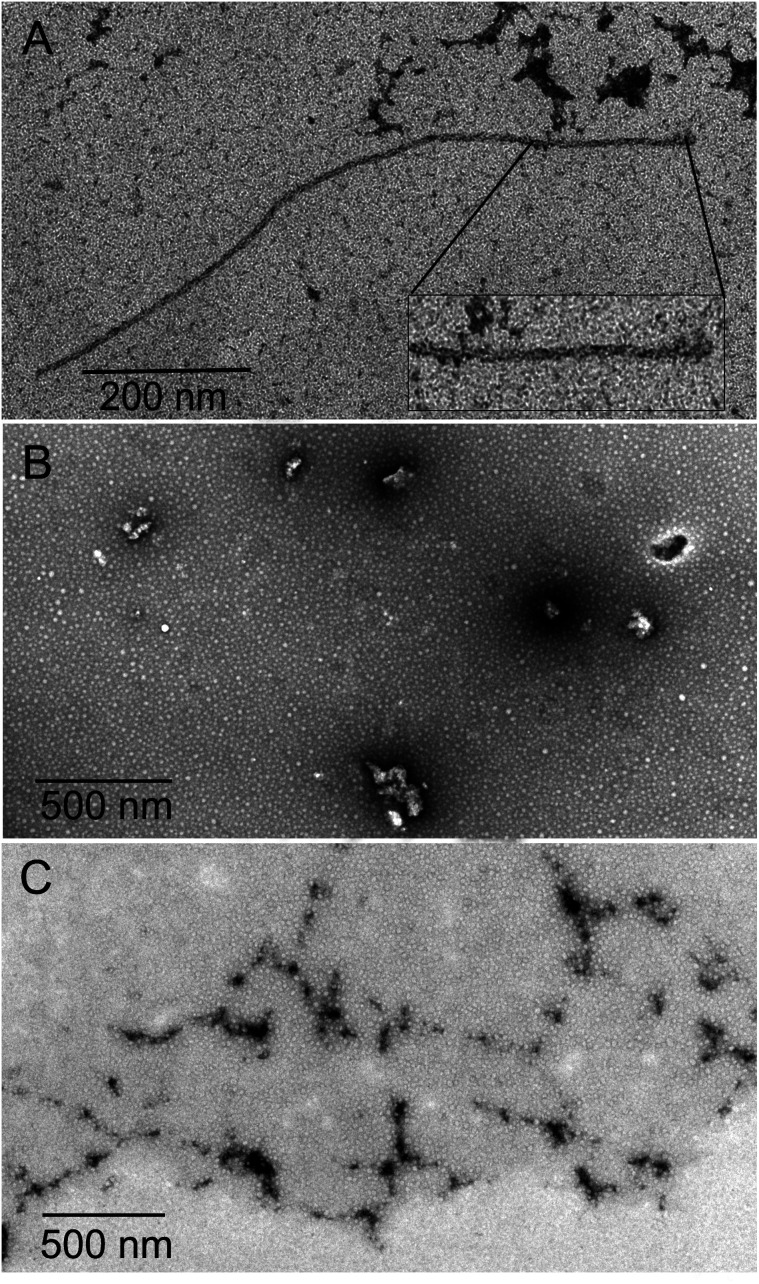
Aβ polymerization assay analysed by TEM. (A) Representative micrograph of identified Aβ polymers. Aβ polymerization assay in the presence of either C_62_(COONa)_4_ or *C*_3_-C_63_(COONa)_6_ (B and C, respectively).

The inhibition of Aβ aggregation by fullerene derivatives has been previously demonstrated; however, it has shown solubility complications in water and high toxicity in cells. In this study, we synthesized adducts of C_60_ with one to three diethylmalonate substituents and their corresponding sodium salts to increase their biocompatibility and capacity to inhibit Aβ aggregation, evaluated by a ThT fluorescence assay. In Fig. 3A, the normalized values of the fluorescence signal of the Aβ aggregates showed significantly lower aggregation of Aβ in the presence of eight different fullerenemalonates compared to the control, in three independent experiments. The highest Aβ aggregation inhibition was shown by both isomeric mixtures of bisadducts, C_62_(COONa)_4_ and *C*_3_-symmetrical trisadduct (*C*_3_-C_63_(COONa)_6_), with 97% and with 80%, respectively (Fig. 3B). To confirm these results, the polymerization assay was analyzed by TEM, showing scattered fibrils (Fig. 4A). In the presence of either C_62_(COONa)_4_ or *C*_3_-C_63_(COONa)_6_ (Fig. 4B and C, respectively) Aβ fibrils were not found. These data indicate that fullerenemalonates inhibit and/or delay Aβ aggregation. The functionalization of C_60_ with two or three disodium malonate substituents increased the efficacy compared to monoaddition.

### Inhibitory activity of the fullerenemalonates by IC_50_

**Fig. 5:**
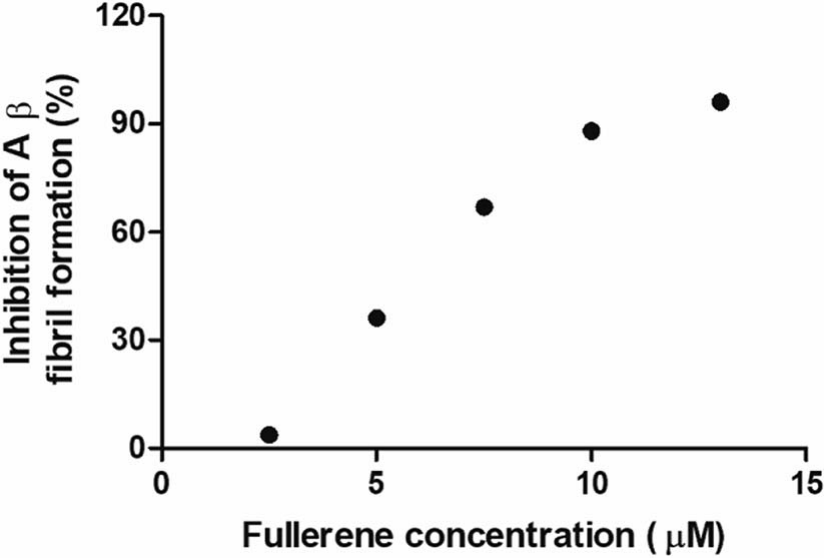
C_62_(COONa)_4_ inhibitory activity on Aβ fibril formation by IC_50_.

To evaluate the Aβ anti-aggregatory capacity of C_62_(COONa)_4_, the most efficient inhibitory fullerene, we determined the IC_50_ value at 24 h by ThT assay, in the concentration range from 2.5 to 13 μM at a fixed peptide concentration of 20 μM. The relative fluorescence spectra showed that C_62_(COONa)_4_ (Fig. 5) inhibits the process of Aβ aggregation in a dose-dependent manner and the concentration to inhibit 50% of Aβ fibril formation determined by IC_50_ value is equal to 6.7 μM.

### Biocompatibility of fullerenemalonates

**Fig. 6:**
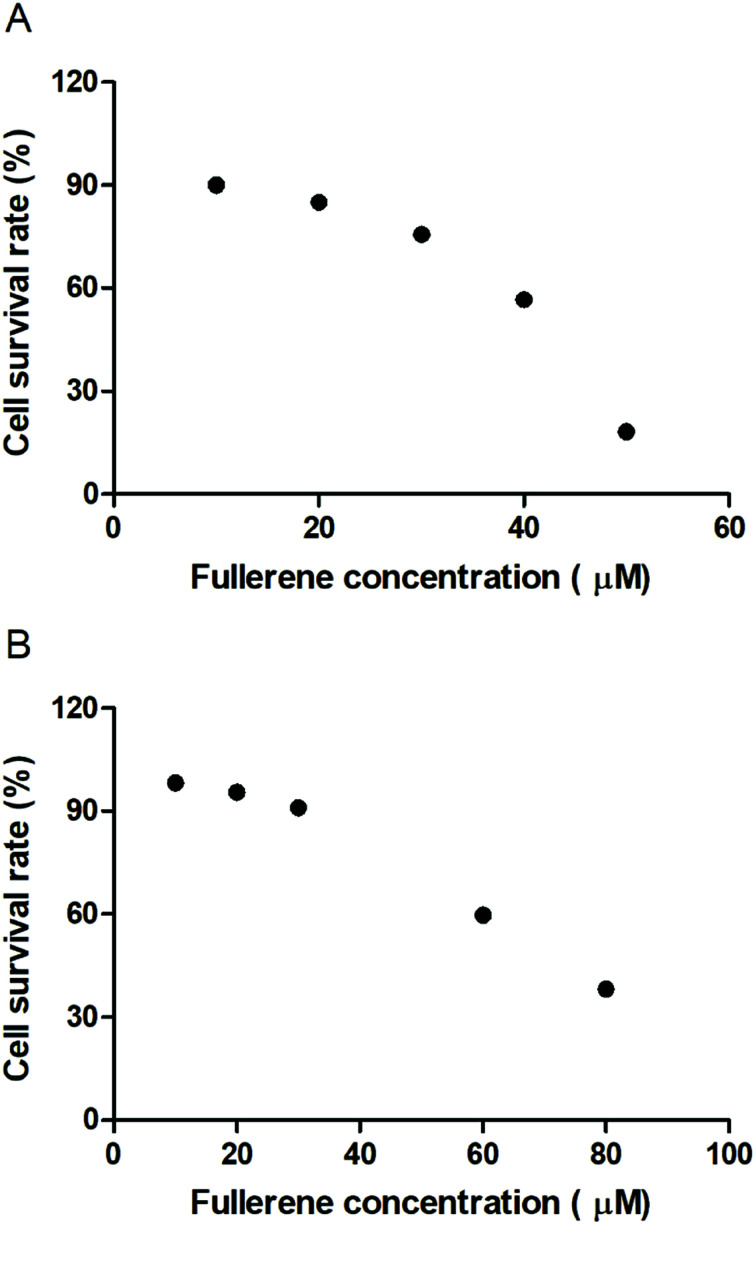
Cytotoxic concentration of C_62_(COONa)_4_ (A) and *C*_3_-C_63_(COONa)_6_ (B) by CC_50_.

To evaluate the cytotoxic effect of the fullerenemalonates, we used the SH-SY5Y cell line that is used as a model for neurodegenerative diseases including AD;^54^ they can be differentiated from a dominantly cholinergic phenotype suitable for AD studies^55^ and they have been tested for Aβ toxicity in a large number of studies.^56–58^ The cells were incubated for 24 h in the presence of fullerenemalonates at 13 μM, corresponding to an Aβ fibril inhibition concentration. The viability of the cells was evaluated by MTT assay. The values normalized to control showed the highest cell viability for fullerenemalonates bearing two (C_62_(COONa)_4_) or three (C_63_(COONa)_6_ and *C*_3_-C_63_(COONa)_6_) disodium malonyl groups (Table 2). Likewise, disodium fullerenemalonates demonstrated higher solubility compared to diethyl fullerenemalonates, indicating that this molecular modification is important for biocompatibility. To determine the cytotoxic concentration of C_62_(COONa)_4_ and *C*_3_-C_63_(COONa)_6_ to reduce cell viability by 50%, CC_50_ was performed (Fig. 6). The dependence of cell viability on fullerene derivative concentration values: CC_50_: 38.8 μM and 69.6 μM for C_62_(COONa)_4_ and *C*_3_-C_63_(COONa)_6_, respectively, confirmed that the toxic concentrations for these molecules are 7 and 12 times higher than the required concentration for Aβ aggregation inhibition.

**Table 2:** Cell viability for fullerenemalonates at 13 μM concentration, using the SH-SY5Y brain-neuroblastoma cell line. Percentage and standard deviation are presented

C_60_ fullerene adducts (13 μM)	Cell survival rate (%)
*C*_3_-C_63_(COONa)_6_	99.34 ± 0.22
C_63_(COONa)_6_ (isomeric mixture)	96.89 ± 0.22
C_62_(COONa)_4_ (isomeric mixture)	91.17 ± 0.44
*C*_3_-C_63_(COOEt)_6_	78.59 ± 0.22
C_63_(COOEt)_6_ (isomeric mixture)	66.43 ± 0.22
C_62_(COOEt)_4_ (isomeric mixture)	66.07 ± 0.08
C_61_(COOEt)_2_	65.65 ± 0.29
C_61_(COONa)_2_	64.34 ± 0.30

### *In silico* interaction of trisadduct with Aβ_42_

**Fig. 7:**
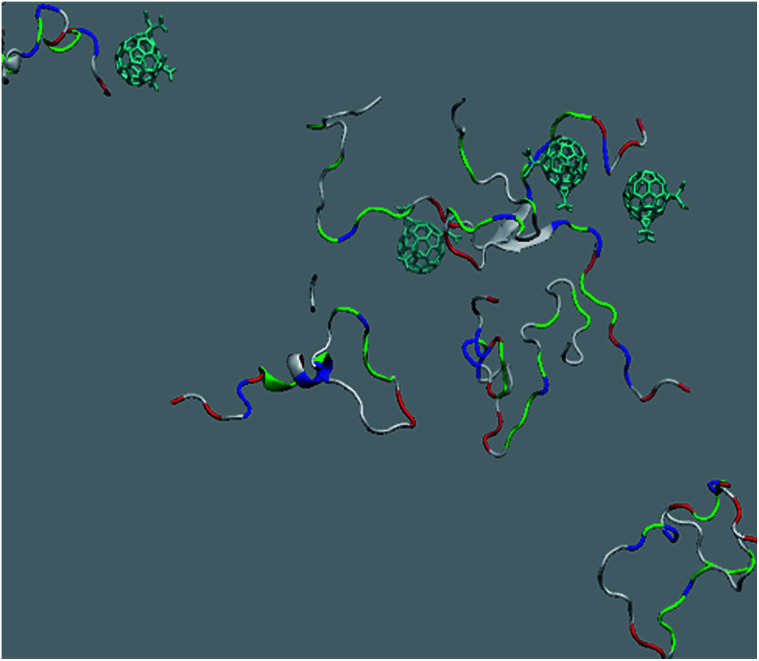
Snapshot of an equilibrated configuration of the ternary system: fullerene trisadduct in blue, Aβ_42_ is represented using different colours for different residues, water molecules are not shown for clarity.

**Fig. 8:**
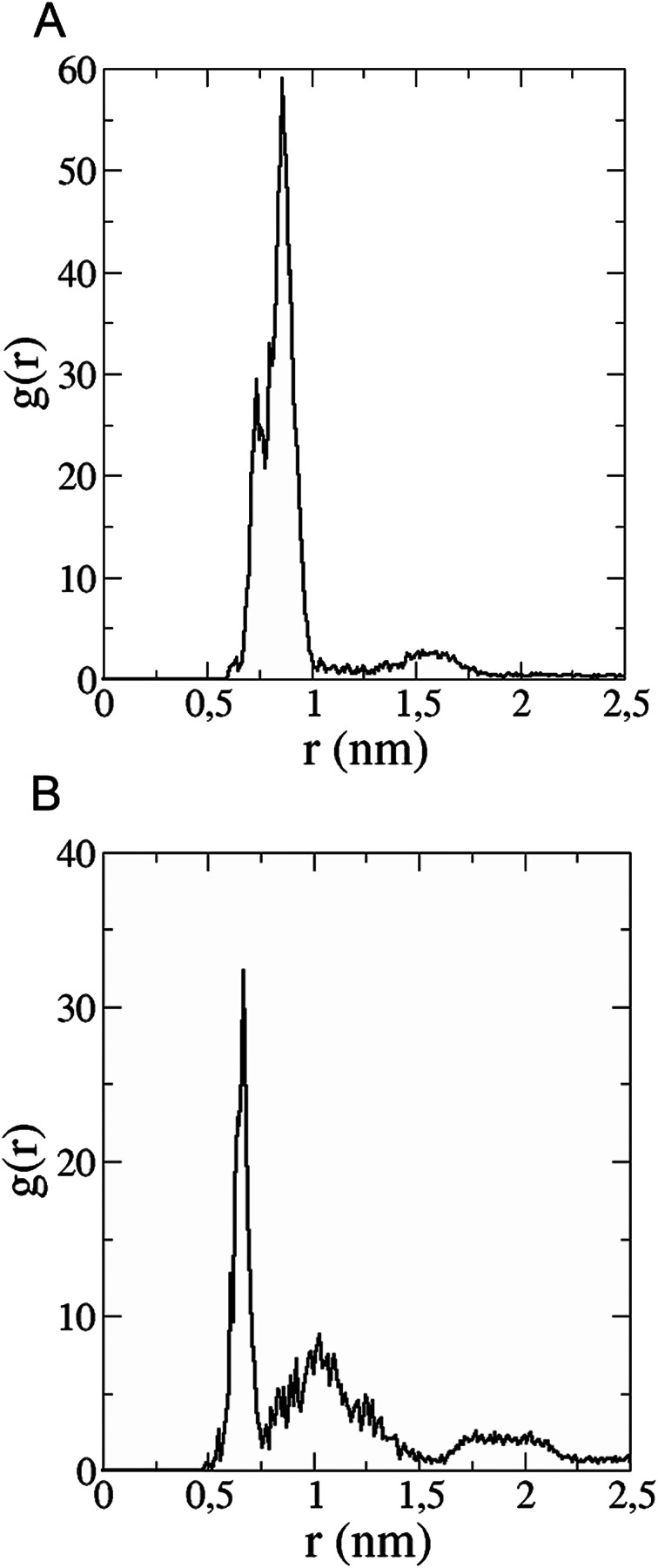
(A) Radial distribution function *versus* distance of 5R residue (Aβ_42_) between centres of mass of residue and fullerene trisadduct. (B) Radial distribution function as a function of the distance between the centre of mass of 1D residue and the two oxygen atoms in the COO-group.

**Fig. 9:**
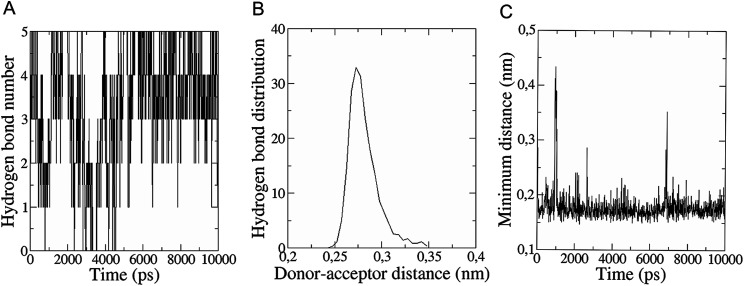
(A) Number of hydrogen bonds during the simulation time between 5R residue (Aβ_42_) and the fullerene trisadduct. (B) Hydrogen bond distribution as a function of time calculated for the 16K residue. (C) Minimal distance during the simulation time between the 16K residue and the fullerene trisadduct using the centres of mass as reference.

The *in vitro* inhibition of the Aβ_42_ fibril formation demonstrated an interaction between the fullerene molecules and Aβ_42_. To propose a mechanism for this effect, the only isolated fullerene, *C*_3_-symmetrical trisadduct (*C*_3_-C_63_(COONa)_6_), was analysed *in silico* in the presence of Aβ_42_ and water to determine the possible interactions leading to inhibition of Aβ_42_ fibril formation. Herein atomistic molecular dynamics simulations of the ternary system were performed and the GROMACS 5.1.4 software package^59^ was used to generate the trajectory files. In Fig. 7 a snapshot of the ternary system can be found; this figure was prepared using the VMD software package^60^ using different colors for different residues; water molecules are not shown for clarity. Radial distribution functions (rdf) were calculated between every residue from Aβ_42_, analysing the center of the mass corresponding to each aminoacid together with that of *C*_3_-C_63_(COONa)_6_. The analysis of the data indicated that residue 1D shows a high pick at 0.83 nm; residue 2A at 0.82 nm; residue 4F has the highest pick at 0.86 nm and a second one at 1.05 nm; 5R has a pick at 0.86 nm, in the same position; residue 6H shows its highest pick at 0.84 nm and a second at 1.16 nm; 8S at 0.88 nm; 12V at 0.82 nm; and finally, residue 15Q shows its highest pick at 0.81 nm. A representative plot of the 5R residue is shown in Fig. 8A. These data suggest a remarkable structure at very short distance (less than 1 nm) between these residues and the fullerene trisadduct, probably binding to the carboxylate group. On average, the fullerene molecules kept their positions during the simulation time. For all the other residues, the picks had an rdf signal lower than 20. The interactions between the residue and the carboxylate group were assessed; the rdf between the center of mass of the two oxygen atoms in the carboxylate group and the center of mass of each residue were also calculated. The analysis of these results indicated that the residues where the rdf was higher than 15 were the 1D, 2A, 4F and 5R residues. Only the plot of the 1D residue is shown in Fig. 8B. 6H, 8S, 12V, 15Q, and 16K residues presented rdf values lower than 20, but they were still well-defined structures. The number of hydrogen bonds and the hydrogen bond distribution as a function of donor–acceptor distance between them and the residues, during the simulation time, were also calculated. The residues that showed hydrogen bonds were 1D, 5R, and 16K. In Fig. 9A and B we show results of the 5R and 16K residues, respectively. 2A and 15Q residues contain a weaker structure of hydrogen bonds, so that they are not retained during the whole simulation time. The minimal distances between each residue and the fullerenemalonate adduct were calculated using the center of mass as reference, and the systems with the shortest distances were the 1D, 5R, and 16K residues. The results for 16K are shown in Fig. 9C. The other residues that retained shorter distances lower than 0.35 nm were 2A, 3E, 4F, 6H, 8S, 11E, 12V, 14H, 15H, 32 G and 35V. These results demonstrated which residues contain strong interactions with *C*_3_-C_63_(COONa)_6_.

## Discussion

In this study, we demonstrated the capacity of fullerenemalonates to inhibit Aβ fibril formation. Our polymerization assay contains mostly monomers at the starting point, which confirms that the interaction of the fullerenes is not directly with pre-aggregates. This is important in terms of the molecular dynamics, since we evaluated the interaction of *C*_3_-C_63_(COONa)_6_ specifically with the Aβ monomer *in silico*. Several experimental and computational studies have demonstrated that the type of substituent inserted on the fullerene surface has a remarkable influence on their anti-amyloid activity. For example, *in vitro* experiments showed that 1,2-(dimethoxymethano) fullerene strongly inhibits Aβ peptide aggregation in the early stages (IC_50_ ∼ 9 μM, Aβ_42_ concentration 20 μM).^16^ Another group has studied the anti-amyloid activity of the sodium salt of the fullerene polycarboxylic derivative C_60_Cl(C_6_H_4_CH_2_COONa), the sodium fullerenolate and complexes of fullerene with polyvinylpyrrolidone and revealed the existence of a strong anti-amyloid activity in Aβ_42_, concluding that the latter two had the most effective Aβ inhibitory effect in both Aβ_42_ and muscle amyloid X-protein.^29,61,62^ Recently, Bednarikova *et al.* demonstrated through *in vitro* and *in silico* experiments that fullerenol, fullerene C_60_ modified with 16 OH groups (C_60_(OH)_16_), inhibits the fibrillization of Aβ_1–40_ in a dose-dependent manner (IC_50_ ∼ 32 μg ml^−1^, Aβ_1–40_ concentration 10 μM) and the inhibition of β-sheet formation results from the strong electrostatic interactions of the fullerenol OH groups with the polar, negatively charged amino acids.^28^ Zhou *et al.* performed multiple all-atom explicit solvent molecular dynamics simulations to study the effect of fullerene substituents and concluded that functionalization with a dimethoxymethane group on the fullerene surface retarded the rotation of the fullerene, thus enhancing the binding stability of the 1,2-(dimethoxymethano)fullerene.^17^ Xie *et al.* found by molecular dynamics simulations that the contact surface area between the fullerenes and the Aβ_16–22_ octamers is an important factor that affects the Aβ–fullerene interaction and a large contact surface area usually implies strong interactions.^63^

The results obtained in this study show that all fullerenemalonates inhibit Aβ_42_ aggregation and their activity depends highly on the number and the nature of the substituents attached to the fullerene surface. In addition, an *in silico* approach demonstrated that the inhibition of Aβ_42_ fibrillization by *C*_3_-C_63_(COONa)_6_ results from strong electrostatic interactions and hydrogen bonding of the fullerenemalonate carboxylate groups predominantly with amino acid groups (residues 1D, 2A, 4F and 5R) and residues 1D, 5R, and 16K, respectively. The higher anti-aggregatory effect of C_62_(COONa)_4_ (98% at 24 h, IC_50_ 6.7 μM, Aβ_42_ concentration 20 μM) may be explained by a balanced relationship between the number of organic addends and the contact surface area available compared to those of the monoadduct and trisadducts. Moreover, disodium fullerenemalonates exhibit higher anti-Aβ activity compared to diethyl fullerenemalonates, which suggests that the addends/surface area ratio, besides the nature of the addends, plays an important role in the anti-Aβ activity of the fullerene derivatives. This finding is consistent with previous studies, suggesting that the strong Aβ–fullerene derivative interaction, due to introduced addends and the contact area available in the fullerene, significantly weakens the Aβ–Aβ interaction and thus inhibits β-sheet formation.^17,28,63^

Likewise, the appended organic addends on the carbon cage in fullerenemalonates make them truly amphiphilic and induce a strong tendency to self-assemble in polar solvents to form stable solutions of nanoaggregates.^25,43,64–68^ Specifically, the self-assembly of sodium carboxylated fullerenes (C_61_(COONa)_2_ and C_62_(COONa)_4_) in aqueous solution produces solid spherical particles with an average hydrodynamic radius *R*_h_ ≈ 32 nm for C_61_(COONa)_2_ and hollow shells with mainly two different size scales of *R*_h_ ≈ 23 nm and *R*_h_ ≈ 104 nm for the isomeric mixture of bisadducts, C_62_(COONa)_4_.^66^ Therefore, it is not surprising that aqueous solutions of disodium fullerenemalonates (C_61_(COONa)_2_, C_62_(COONa)_4_ and C_63_(COONa)_6_) and other water-soluble fullerene derivatives evaluated as inhibitors of Aβ aggregation comprise nanoclusters rather than individual solvated molecules.^16,24,25,29,30,61,62,69^ Nevertheless, the results obtained in this study and other reports show that self-assembly in polar solvents to form stable nanoaggregates does not affect its anti-Aβ activity.

Regarding the biocompatibility of fullerenemalonates, our results showed that modification of the fullerene surface with diethyl malonyl groups causes higher cytotoxicity compared to those with disodium malonyl groups. Moreover, it was found that, excluding the monoadduct sodium salt (C_61_(COONa)_2_), disodium fullerenemalonates with two or three substituents were not toxic for the SH-SY5Y neuroblastoma cell line at the half maximal inhibitory concentration (IC_50_ 6.7 μM). Likewise, it was found that the cytotoxicity is reduced as the number of disodium malonyl substituents attached to the fullerene surface and their attachment symmetry increase. The higher biocompatibility of *C*_3_-symmetrical triadduct (*C*_3_-C_63_(COONa)_6_, viability 99%) compared to those of monoadduct (C_61_(COONa)_2_, viability 64%) and the isomeric mixture of bisadducts (C_62_(COONa)_4_, viability 91%) and triadducts (C_63_(COONa)_6_, viability 97%) may be attributed to the combined effect of the decrease in hydrophobic surface due to hydrophilic addends attached to the fullerene core and the *e*,*e*,*e*-symmetrical addition pattern of *C*_3_-C_63_(COONa)_6_. This study agrees with the reported literature, which suggests that a higher abundance of hydrophilic addends on the fullerene surface and a high-symmetry addition pattern, result in a decrease in cytotoxicity.^29,70,71^

## Conclusions

In this study we have demonstrated that fullerenemalonates bearing 1 to 3 diethyl malonyl substituents and their corresponding sodium salts interact and effectively reduce Aβ fibril formation *in vitro*. The bisadduct salts (C_62_(COONa)_4_) and trisadduct (C_63_(COONa)_6_) inhibit 98 and 83% of Aβ aggregation, respectively. The 6.7 μM IC_50_ value of a C_62_(COONa)_4_ mixture confirmed one of the highest anti-amyloid capacities that has been reported. The anti-aggregatory effect of the bisadduct salts, C_62_(COONa)_4_, is mostly attributed to the balance between the hydrophobic surface and the number of substituents bound to the fullerene, promoting stability in the interaction with the Aβ peptide. The sodium salts C_62_(COONa)_4_, C_63_(COONa)_6_ and *C*_3_-C_63_(COONa)_6_ showed low toxicity in neuroblastoma SH-SY5Y cell viability, suggesting that these molecules are highly biocompatible at concentrations which are able to effectively inhibit Aβ aggregation. The lowest toxicity presented in the trisadduct salt, *C*_3_-C_63_(COONa)_6_, is associated with the combined effect of the reduction in the fullerene hydrophobic surface through the addition of hydrophilic substituents and the fullerene symmetry. The effective anti-amyloid activity and low toxicity of the bisadduct isomeric mixture (C_62_(COONa)_4_) and trisadduct (C_63_(COONa)_6_) could be promising candidates for further animal studies, and potential therapeutic molecules for the treatment of Alzheimer's disease.

## Conflicts of interest

There are no conflicts to declare.

## Supplementary Material

## References

[cit1] Hardy J. (1997). Trends Neurosci..

[cit2] Mucke L., Masliah E., Yu G. Q., Mallory M., Rockenstein E. M., Tatsuno G., Hu K., Kholodenko D., Johnson-Wood K., McConlogue L. (2000). J. Neurosci..

[cit3] Klein W. L., Krafft G. A., Finch C. E. (2001). Trends Neurosci..

[cit4] Jarrett J. T., Lansbury Jr P. T. (1993). Cell.

[cit5] Kumar S., Walter J. (2011). Aging.

[cit6] Orgogozo J. M., Gilman S., Dartigues J. F., Laurent B., Puel M., Kirby L. C., Jouanny P., Dubois B., Eisner L., Flitman S., Michel B. F., Boada M., Frank A., Hock C. (2003). Neurology.

[cit7] Uro-Coste E., Russano de Paiva G., Guilbeau-Frugier C., Sastre N., Ousset P. J., da Silva N. A., Lavialle-Guillotreau V., Vellas B., Delisle M. B. (2010). Clin. Neuropathol..

[cit8] Sisodia S. S., St George-Hyslop P. H. (2002). Nat. Rev. Neurosci..

[cit9] Fuse S., Matsumura K., Fujita Y., Sugimoto H., Takahashi T. (2014). Eur. J. Med. Chem..

[cit10] Nakagami Y., Nishimura S., Murasugi T., Kaneko I., Meguro M., Marumoto S., Kogen H., Koyama K., Oda T. (2002). Br. J. Pharmacol..

[cit11] O'Hare E., Scopes D. I., Treherne J. M., Norwood K., Spanswick D., Kim E. M. (2010). Behav. Brain Res..

[cit12] Baig M. H., Ahmad K., Rabbani G., Choi I. (2018). Front. Aging Neurosci..

[cit13] Viet M. H., Siposova K., Bednarikova Z., Antosova A., Nguyen T. T., Gazova Z., Li M. S. (2015). J. Phys. Chem. B.

[cit14] Sciarretta K. L., Gordon D. J., Meredith S. C. (2006). Methods Enzymol..

[cit15] Bellova A., Bystrenova E., Koneracka M., Kopcansky P., Valle F., Tomasovicova N., Timko M., Bagelova J., Biscarini F., Gazova Z. (2010). Nanotechnology.

[cit16] Kim J. E., Lee M. (2003). Biochem. Biophys. Res. Commun..

[cit17] Zhou X., Xi W., Luo Y., Cao S., Wei G. (2014). J. Phys. Chem. B.

[cit18] Yang Z., Ge C., Liu J., Chong Y., Gu Z., Jimenez-Cruz C. A., Chai Z., Zhou R. (2015). Nanoscale.

[cit19] Friedman S. H., DeCamp D. L., Sijbesma R. P., Srdanov G., Wudl F., Kenyon G. L. (1993). J. Am. Chem. Soc..

[cit20] Xiao L., Aoshima H., Saitoh Y., Miwa N. (2011). Free Radic. Biol. Med..

[cit21] Meng J., Liang X., Chen X., Zhao Y. (2013). Integr. Biol..

[cit22] Markovic Z., Trajkovic V. (2008). Biomaterials.

[cit23] Tanimoto S., Sakai S., Matsumura S., Takahashi D., Toshima K. (2008). Chem. Commun..

[cit24] Ishida Y., Fujii T., Oka K., Takahashi D., Toshima K. (2011). Chem.–Asian J..

[cit25] Hasunuma N., Kawakami M., Hiramatsu H., Nakabayashi T. (2018). RSC Adv..

[cit26] Du Z., Gao N., Wang X., Ren J., Qu X. (2018). Small.

[cit27] Raoof M., Mackeyev Y., Cheney M. A., Wilson L. J., Curley S. A. (2012). Biomaterials.

[cit28] Bednarikova Z., Huy P. D., Mocanu M. M., Fedunova D., Li M. S., Gazova Z. (2016). Phys. Chem. Chem. Phys..

[cit29] Bobylev A. G., Kornev A. B., Bobyleva L. G., Shpagina M. D., Fadeeva I. S., Fadeev R. S., Deryabin D. G., Balzarini J., Troshin P. A., Podlubnaya Z. A. (2011). Org. Biomol. Chem..

[cit30] Ishida Y., Tanimoto S., Takahashi D., Toshima K. (2010). MedChemComm.

[cit31] Johnson-Lyles D. N., Peifley K., Lockett S., Neun B. W., Hansen M., Clogston J., Stern S. T., McNeil S. E. (2010). Toxicol. Appl. Pharmacol..

[cit32] Su Y., Xu J. Y., Shen P., Li J., Wang L., Li Q., Li W., Xu G. T., Fan C., Huang Q. (2010). Toxicology.

[cit33] Kraemer A. B., Parfitt G. M., Acosta D. D. S., Bruch G. E., Cordeiro M. F., Marins L. F., Ventura-Lima J., Monserrat J. M., Barros D. M. (2018). Toxicol. Appl. Pharmacol..

[cit34] Bingel C. (1993). Chem. Ber..

[cit35] Billups W. E. (2005). J. Am. Chem. Soc..

[cit36] Camps X., Hirsch A. (1997). J. Chem. Soc., Perkin Trans. 1.

[cit37] Straface E., Natalini B., Monti D., Franceschi C., Schettini G., Bisaglia M., Fumelli C., Pincelli C., Pellicciari R., Malorni W. (1999). FEBS Lett..

[cit38] Hirsch A., Lamparth I., Karfunkel H. R. (1994). Angew. Chem., Int. Ed. Engl..

[cit39] Djojo F., Hirsch A., Grimme S. (1999). Eur. J. Org. Chem..

[cit40] Hirsch A., Lamparth I., Groesser T., Karfunkel H. R. (1994). J. Am. Chem. Soc..

[cit41] Martínez-Herrera M., Amador P., Rojas A. (2011). J. Phys. Chem. C.

[cit42] Lu Q., Schuster D. I., Wilson S. R. (1996). J. Org. Chem..

[cit43] Guldi D. M., Hungerbuehler H., Janata E., Asmus K. D. (1993). J. Phys. Chem..

[cit44] Zhang-Haagen B., Biehl R., Nagel-Steger L., Radulescu A., Richter D., Willbold D. (2016). PLoS One.

[cit45] Stine Jr W. B., Dahlgren K. N., Krafft G. A., LaDu M. J. (2003). J. Biol. Chem..

[cit46] Stine W. B., Jungbauer L., Yu C., LaDu M. J. (2011). Methods Mol. Biol..

[cit47] Naiki H., Higuchi K., Hosokawa M., Takeda T. (1989). Anal. Biochem..

[cit48] Crescenzi O., Tomaselli S., Guerrini R., Salvadori S., D'Ursi A. M., Temussi P. A., Picone D. (2002). Eur. J. Biochem..

[cit49] Paulus E. F., Bingel C. (1995). Acta Crystallogr., Sect. C: Cryst. Struct. Commun..

[cit50] Malde A. K., Zuo L., Breeze M., Stroet M., Poger D., Nair P. C., Oostenbrink C., Mark A. E. (2011). J. Chem. Theory Comput..

[cit51] Huang J., MacKerell Jr A. D. (2013). J. Comput. Chem..

[cit52] Jorgensen W. L., Chandrasekhar J., Madura J. D. (1983). J. Chem. Phys..

[cit53] MacKerell A. D., Bashford D., Bellott M., Dunbrack R. L., Evanseck J. D., Field M. J., Fischer S., Gao J., Guo H., Ha S., Joseph-McCarthy D., Kuchnir L., Kuczera K., Lau F. T. K., Mattos C., Michnick S., Ngo T., Nguyen D. T., Prodhom B., Reiher W. E., Roux B., Schlenkrich M., Smith J. C., Stote R., Straub J., Watanabe M., Wiórkiewicz-Kuczera J., Yin D., Karplus M. (1998). J. Phys. Chem. B.

[cit54] Xicoy H., Wieringa B., Martens G. J. (2017). Mol. Neurodegener..

[cit55] Edsjo A., Lavenius E., Nilsson H., Hoehner J. C., Simonsson P., Culp L. A., Martinsson T., Larsson C., Pahlman S. (2003). Lab. Invest..

[cit56] Datki Z., Juhasz A., Galfi M., Soos K., Papp R., Zadori D., Penke B. (2003). Brain Res. Bull..

[cit57] Milton N. G., Chilumuri A., Rocha-Ferreira E., Nercessian A. N., Ashioti M. (2012). ACS Chem. Neurosci..

[cit58] Krishtal J., Bragina O., Metsla K., Palumaa P., Tougu V. (2017). PLoS One.

[cit59] Humphrey W., Dalke A., Schulten K. (1996). J. Mol. Graphics.

[cit60] Abraham M. J., Murtola T., Schulz R., Páll S., Smith J. C., Hess B., Lindahl E. (2015). SoftwareX.

[cit61] Bobylev A. G., Marsagishvili L. G., Podlubnaia Z. A. (2010). Biofizika.

[cit62] Bobylev A. G., Shpagina M. D., Bobyleva L. G., Okuneva A. D., Piotrovskii L. B., Podlubnaia Z. A. (2012). Biofizika.

[cit63] Xie L., Luo Y., Lin D., Xi W., Yang X., Wei G. (2014). Nanoscale.

[cit64] Angelini G., De Maria P., Fontana A., Pierini M., Maggini M., Gasparrini F., Zappia G. (2001). Langmuir.

[cit65] Partha R., Lackey M., Hirsch A., Casscells S. W., Conyers J. L. (2007). J. Nanobiotechnol..

[cit66] Zhou S. Q., Ouyang J. Y., Golas P., Wang F., Pan Y. (2005). J. Phys. Chem. B.

[cit67] Georgakilas V., Pellarini F., Prato M., Guldi D. M., Melle-Franco M., Zerbetto F. (2002). Proc. Natl. Acad. Sci. U. S. A..

[cit68] Hsieh F. Y., Zhilenkov A. V., Voronov II, Khakina E. A., Mischenko D. V., Troshin P. A., Hsu S. H. (2017). ACS Appl. Mater. Interfaces.

[cit69] Zha Y. Y., Yang B., Tang M. L., Guo Q. C., Chen J. T., Wen L. P., Wang M. (2012). Int. J. Nanomed..

[cit70] Andrievsky G., Klochkov V., Derevyanchenko L. (2005). Fullerenes, Nanotubes, Carbon Nanostruct..

[cit71] Sayes C. M., Fortner J. D., Guo W., Lyon D., Boyd A. M., Ausman K. D., Tao Y. J., Sitharaman B., Wilson L. J., Hughes J. B., West J. L., Colvin V. L. (2004). Nano Lett..

